# *Lactobacillus plantarum* Strains Isolated from Polish Regional Cheeses Exhibit Anti-Staphylococcal Activity and Selected Probiotic Properties

**DOI:** 10.1007/s12602-019-09587-w

**Published:** 2019-08-28

**Authors:** Aleksandra Ołdak, Dorota Zielińska, Anna Łepecka, Ewa Długosz, Danuta Kołożyn-Krajewska

**Affiliations:** 1 Department of Food Gastronomy and Food Hygiene, Faculty of Human Nutrition and Consumer Sciences, Warsaw University of Life Science – SGGW, Nowoursynowska 159c, 02-776 Warsaw, Poland; 2 Department of Preclinical Sciences, Faculty of Veterinary Medicine, Warsaw University of Life Science – SGGW, Ciszewskiego 8, 02-776 Warsaw, Poland

**Keywords:** Anti-staphylococcal activity, *Lactobacillus plantarum*, Protective cultures, Regional cheese

## Abstract

Twenty-nine *Lactobacillus plantarum* strains isolated from different types of Polish regional cheeses (Oscypek and Korycinski) were assessed for selected probiotic properties and anti-staphylococcal activity. Most of the tested *L. plantarum* strains were considered safe. Whole bacterial cultures (WBC) and cell-free supernatants (CFSs) of *L. plantarum* strains inhibited growth of *Staphylococcus aureus* (average inhibition growth zones were 2.8 mm ± 1.2 and 2.8 mm ± 1.1 respectively). Moreover, almost all neutralized, catalase-treated cell-free supernatants (CFN) of *L. plantarum* cultures also exhibited slight anti-staphylococcal activity in vitro. The most promising strains Os4 and Kor14 were selected for further study. Both strains were able to survive during digestive gastro-intestinal passage model. Live cells of *L. plantarum* Os4 and Kor14 caused the strongest inhibition of *S. aureus* adhesion to Caco-2 cells comparing with CFN and heat-killed bacterial cells. *S. aureus* and *L. plantarum* (Os4 or Kor14) co-cultured in skim milk resulted in growth inhibition of *S. aureus* in both 8 °C and 37 °C incubation temperatures*.* Observed abilities, demonstrated for *L. plantarum* Os4 and Kor14, confirms that these strains could be used in the food industry as protective cultures.

**Electronic supplementary material:**

The online version of this article (10.1007/s12602-019-09587-w) contains supplementary material, which is available to authorized users.

## Introduction

Lactic acid bacteria (LAB) are ubiquitous microorganisms that give food-specific functional and sensory properties. Furthermore, numerous studies indicate that their presence positively affects the human health by colonizing the digestive tract, lowering of blood cholesterol, or competing with pathogens [1]. Traditional food in different regions of the world is a particularly rich source of LAB, which is connected with the natural process of production and often the spontaneous fermentation reaction. LAB in traditional foods are responsible for the flavor characteristics as well as prevent the development of undesirable microbes, which directly prolongs the sustainability and preservation of the microbiological safety of the product [2].

*Staphylococcus aureus* is a Gram-positive bacterium commonly found on the skin and also in the nasopharynx of humans and animals. In a food product, this bacterium produces heat-stable enterotoxin, in which consumption of this causes the symptoms of intoxication such as diarrhea, abdominal pain, nausea, and fever [3, 4].

Staphylococcal intoxication is often connected with the consumption of cheeses. Only in 2015 in the EU, it was observed that cheese was the vehicle of 33% of strong-evidence outbreaks [5]. Overall, in 2015, 39 strong-evidence outbreaks and 395 weak-evidence ones were reported. Moreover, a high rate of hospitalization was observed (113 patients hospitalized per 758 overall number of cases) in strong-evidence outbreaks. It shows that the special attention should be paid on presence of *S. aureus* in food products, mainly in raw milk cheeses, because of the high risk for food safety [5].

Numerous studies show that LAB isolated from different kinds of food can effectively inhibit the growth of *S. aureus* [2, 6–9]. Anti-bacterial activity of LAB is mainly connected with the pH lowering and organic acid production and also with the possibility of bacteriocin synthesis and other antimicrobial agents such as hydrogen peroxide, reuterin or reutericyclin, and peptidoglycan hydrolases [10]. These LAB cultures which show unique features could be possibly used in food technology in the future, especially when chemical preservation is undesirable. It has been already shown that LAB isolated from Polish regional cheeses exhibit strong anti-bacterial activity against foodborne pathogens such as *Listeria monocytogenes*, *Salmonella enteritidis*, and *Escherichia coli* [11, 12]. If they also inhibit the growth of *S. aureus* and survive in gastro-intestinal tract to trigger a positive impact for the consumer, it would be possible to use these strains as probiotic and protective cultures.

The aim of this study was the evaluation of anti-staphylococcal activity, as well as preliminary safety assessment of *Lactobacillus plantarum* strains isolated from Polish regional cheeses and recognition the possibilities of using these strains as a probiotic and protective cultures in food technology. The strains were screened in vitro for their anti-staphylococcal activity and the selection was made. The best strains were analyzed to check their ability to survive in human gastro-intestinal tract and compete for in vitro adhesion to human gut cells as well as anti-staphylococcal activity in food matrix.

## Material and Methods

### Strains and Culture Conditions

The 29 strains of *L. plantarum* were isolated and genetically identified in the previous study of Ołdak et al. [11]. Strain cultures were stored at – 80 °C with 20% glycerol (v/v) addition in MRS broth (LabM, UK) and the fresh cultures were made prior the assays. The probiotic strain *L. plantarum* 299v was used as a reference.

*S. aureus* ATCC 25923 and *S. aureus* 12.21 and 4.4—food origin pathogens—were used as the indicator strains. The strains *S. aureus* 12.21 and 4.4 were kindly provided by the collection of Chair of Industrial and Food Microbiology, University of Warmia and Mazury, Poland.

To determine the count of *S. aureus*, serial decimal dilutions were plated onto Baird-Parker Agar supplemented with the Egg Yolk Tellurite (Merck, Germany). Enumeration of *L. plantarum* was performed on MRS agar (LabM, UK). Chosen media were selective for mentioned microorganisms used in this study. The plates were incubated for 24–48 h at 37 °C. After incubation, colonies observed on each solid medium were counted and results were converted to logarithmic scale.

### Antibiotic Resistance

The *L. plantarum* strains were analyzed to evaluate their resistance to the most common antibiotics using the E-test (HiMedia, India) in accordance with the manual provided by the producer. To determine the minimal concentration of antibiotic (MIC) inhibiting the growth of the microorganism, the inhibition zones were checked, and the antibiotic concentration were read from strips. Bacterial strains were categorized as susceptible or resistant to antimicrobials according to EFSA Scientific Opinion [14]:

- Susceptible (S): a bacterial strain is defined as susceptible when it is inhibited at a concentration of a specific antimicrobial equal or lower than the established cut-off value (S ≤ *x* mg/l).
- Resistant (R): a bacterial strain is defined as resistant when it is not inhibited at a concentration of a specific antimicrobial higher than the established cut-off value (R > *x* mg/l)

The susceptibility or resistance were evaluated to gentamycin (0.016–256 μg/ml), streptomycin (0.016–256 μg/ml), ampicillin (0.016–256 μg/ml), vancomycin (0.016–256 μg/ml), tetracycline (0.016–256 μg/ml), ciprofloxacin (0.016–256 μg/ml), chloramphenicol (0.016–256 μg/ml), kanamycin (0.016–256 μg/ml), penicillin (0.002–32 μg/ml), and erythromycin (0.016–256 μg/ml). Each time, 200 μl of culture in MRS broth medium (0.5 McFarland standard) was inoculated on Petri plates with Mueller-Hinton agar (LabM, UK). Then, the antibiotic strips were placed. Strains were incubated at 37 °C for 48 h under anaerobic conditions in the GENBox anaer (bioMérieux, France). The results were compared with MIC value suggestions of Danielsen and Wind [13, 14].

### Enzymatic Profile

Enzymatic profiles of the *L. plantarum* strains were determined using API ZYM (bioMerieux, France) in accordance with the manufacturer’s protocols. Strains were incubated at 37 °C for 24 h. Five groups of enzyme activities were analyzed: lipolytic activity (including enzyme activity of esterase, esterase lipase, lipase), proteolytic activity (including enzyme activity of leucine arylamidase, valine arylamidase, cysteine arylamidase, trypsin, and α-chymotrypsin), saccharolytic activity (including enzyme activity of α-galactosidase, β-galactosidase, α-glucosidase, β-glucosidase, α-mannosidase, and α-fucosidase), phosphatase activity (including enzyme activity of alkaline phosphatase, acid phosphatase, naphthol-AS-BI-phosphohydrolase), and harmful enzyme activity (production of α-chymotrypsin, β-glucuronidase and *N*-acetyl-β-glucosaminidase).

### Screening for Anti-Staphylococcal Activity

Anti-staphylococcal effectiveness of whole bacterial cultures (WBC), cell-free supernatants (CFS), and neutralized, catalase-treated cell-free supernatants (CFN) of the 29 *L. plantarum* strains were screened using well diffusion method (each in triplicate). Briefly, the 24 h cultures (in stationary phase of growth) were centrifuged (6000×*g*, 10 min) and sterilized by filtration (0.2 μm) and the anti-staphylococcal activities of CFS were tested. Subsequently, the obtained supernatants were treated with catalase (300 IU/ml, Sigma Aldrich, Poland) and neutralized with 1 M NaOH for the exclusion of the influence of organic acids, lowered pH, or hydrogen peroxide synthesis on the antagonistic activity of tested *L. plantarum* strains.

Sterile Petri dishes were aseptically poured with Mueller-Hinton Agar (LabM, UK), dried, and inoculated with 200-μl culture of each *S. aureus* strain (concentration 6 log CFU/ml, measured by plate count method). After that, 5.5-mm-diameter wells were cut out and subsequently filled with 50 μl of WBC, CFS, and CFN. Afterwards, plates were incubated at 37 °C for 48 h and zones of growth inhibition were observed and measured. Anti-staphylococcal activity of WBC and CFS (*x*) was converted as follows: *x* = *D* − *d*, where *D* is the zone diameter and *d* is the cut well diameter. Antagonistic effect of CFN was, in turn, evaluated qualitatively as “+” which means slight (< 1 mm of diameter inhibition zone) and “−” which means none.

Two strains showing the highest overall anti-staphylococcal activity, *L. plantarum* Os4 isolated from Oscypek and *L. plantarum* Kor14 isolated from Korycinski cheese, were chosen to further studies.

### Survival Ability in the Gastro-Intestinal Tract

Gastric and intestinal juices were prepared according to Rzepkowska et al. [15] and suggestions of Minekus et al. [16]. Gastric juice consisted of the following: distilled water, NaCl (5 g/l), and pepsin (3 g/l) adjusted to pH 2.0 with HCl, whereas intestinal juice consisted of the following: distilled water, NaHCO_3_ (6.4 g/l), KCl (0.239 g/l), NaCl (1.28 g/l), bile salts (3.0 g/l), and pancreatin (1.0 g/l), adjusted to pH 8.0 with NaOH.

Briefly, 1 ml overnight culture of *L. plantarum* (concentration approx. 10^9^–10^10^ CFU/ml) was centrifuged 10,000×*g* for 5 min. Therefore, supernatant was removed and 5 ml of gastric juice was added. Samples were vortexed and incubated for 2 h at 37 °C. After incubation period, samples were centrifuged (10,000×*g*/5 min.), supernatant was removed, and 10 ml of intestinal juice was added. Samples were vortexed and additionally incubated for 3 h at 37 °C. *L. plantarum* count was assessed before (0 h) and after incubation in gastric juice (2 h) and after incubation in gastric and intestinal juice (5 h) by a plate count technique. All tests were done in triplicate.

### Competition for Adhesion to Caco-2 Cells

Caco-2 epithelial cells, originating from human colorectal adenocarcinoma (ATCC HTB-37), were used in their terminally differentiated state to mimic small intestine mature enterocytes. The cells were cultured in Dulbecco’s modified Eagle’s minimal essential medium (DMEM, Biowest) supplemented with 10% (v/v) heat-inactivated fetal bovine serum (Biowest), 2 mM l-glutamine (Biowest), 100 U/ml penicillin, and 100 mg/ml streptomycin (Biowest) at 37 °C in an atmosphere containing 5% CO_2_. The culture medium was changed every other day and replaced by fresh DMEM. Cells were used between 26 and 40 cell passages.

The adherence of tested strains to Caco-2 cell line was tested according to Delgado et al. [17] with slight modifications. Caco-2 cells were seeded in 24-well tissue culture plates at 5 × 10^5^ cells per well and grown during 14 days to obtain a monolayer of differentiated and polarized cells. The culture medium was changed every 2 days. Bacterial cultures (1 ml) were grown until they reached the late exponential phase and they were sedimented by centrifugation (10,000×*g* for 10 min). Then, they were washed twice and resuspended in fresh DMEM at a concentration of 10^8^ CFU/ml and added to each well of the tissue culture plate containing a Caco-2 monolayer. In competition tests, *S. aureus* ATCC 25923 and *L. plantarum* Os4 or Kor 14 were co-cultured in 1:1 ratio. *L. plantarum* were used as WBC or as heat-inactivated (80 °C per 20 min) bacterial cultures (HK). Addition of *L. plantarum* CFNs (100 μl per well) was also tested as a competition factor. The plates were incubated for 2 h at 37 °C in a 5% CO_2_ atmosphere. After incubation, unattached bacteria were removed by washing the monolayers four times with sterile PBS. Additionally, 200 μl of trypsin (2.5%, *w*/*v*) was added to detach the monolayer. The cells were incubated for 15 min at room temperature and recovered by repeated pipetting with 800 μl of sterile ultra-pure water and bacteria were enumerated by plating serial dilutions on solid medium. Adhesion values (%) were calculated as follows:

$$ \%\mathrm{Adhesion}=\left({V}_1\times 100\right)/{V}_0 $$

where *V*_0_ is the initial viable count of bacteria tested, and *V*_1_ is the viable bacterial count obtained from Caco-2 cells, at the end of the experiment.

Each adhesion assay was conducted in triplicate.

### Co-Culture in Skim Milk

Considering that *L. plantarum* strains were isolated from dairy products, the assessment of their anti-staphylococcal activity in milk matrix was conducted.

Ten milliliters of skim milk (Mlekovita, Poland) was inoculated with ~ 10^2^ cells of indicator strain (*S. aureus* ATCC 25923). Afterwards, ~ 10^6^ bacterial cells of *L. plantarum* (Os4 or Kor14) were added as WBC. Control samples were inoculated only with the indicator strain. Milk samples were incubated at 37 °C and 8 °C for 24 h, 72 h, and 120 h. The initial bacterial count and the enumeration in each time point were performed by a plate count technique. Each experiment was conducted in triplicate.

### Statistical Analysis

Data are expressed as a mean ± standard error (SE) calculated over three independent experiments performed in triplicate. Analysis of statistical significance was performed by ANOVA with Student’s *t* test. Statistical analyses were performed using STATISTICA13PL (StatSoft Inc.).

## Results

### Antibiotic Resistance

Table 1 shows the number of *L. plantarum* strains isolated from regional cheeses that have been considered as resistant to antibiotics. Most of the tested strains were susceptible to gentamicin, ampicillin, tetracycline, chloramphenicol, and kanamycin. Total resistance of tested strains to streptomycin and vancomycin was demonstrated. Almost all bacterial strains isolated from the Oscypek were resistant to ciprofloxacin, which was less noticeable in the strains isolated from the Korycinski cheese. On the other hand, resistance to penicillin and erythromycin was found among the strains isolated from Korycinski cheese.

**Table 1:** Antibiotic susceptibility or resistance of *Lactobacillus* strains

	Type of antibiotic									
Strain symbol	GN^1^	ST^1^	AM^1^	VA^1^	TR^1^	CPH^2^	CHL^1^	KN^1^	PN^2^	ER^1^
Os1	S	R	R	R	S	S	S	S	R	S
Os2	S	R	R	R	S	R	S	R	R	S
Os3	S	R	S	R	S	R	S	S	R	S
Os4	S	R	S	R	S	R	S	S	R	S
Os5	S	R	S	R	S	R	R	S	R	R
Os6	S	R	S	R	S	R	R	S	S	R
Os7	S	R	S	R	S	R	R	S	S	S
Os8	S	R	S	R	S	R	S	S	R	S
Os9	S	R	S	R	S	R	R	S	S	S
Os10	S	R	S	R	S	R	S	S	R	S
Os11	S	R	S	R	S	R	S	S	S	R
Os12	S	R	S	R	S	R	S	S	R	R
Os13	S	R	S	R	S	R	R	S	S	S
Os14	S	R	S	R	S	R	S	S	S	S
Os15	S	R	S	R	S	R	S	R	R	S
Os16	S	R	S	R	S	R	S	S	S	R
Os17	S	R	S	R	S	R	R	S	S	R
Kor1	S	R	S	R	R	S	S	S	R	R
Kor2	S	R	S	R	S	R	R	S	R	R
Kor8	S	R	R	R	R	S	S	S	R	R
Kor11	S	R	S	R	S	S	R	S	R	R
Kor12	S	R	R	R	S	S	S	S	R	R
Kor13	S	R	S	R	S	S	S	S	R	R
Kor14	S	R	S	R	R	S	S	S	R	R
Kor15	S	R	S	R	R	R	S	S	R	R
Kor18	S	R	S	R	S	R	S	S	R	R
Kor19	S	R	S	R	R	R	S	S	R	S
Kor22	S	R	R	R	R	R	S	S	R	R
Kor23	S	R	S	R	S	R	S	S	R	S

^1^MIC according to EFSA (2012)

^2^MIC according to Danielsen and Wind (2003)

*R* resistant, *S* susceptible, *GN* gentamicin, *ST* streptomycin, *AM* ampicillin, *VA* vancomycin, *TR* tetracycline, *CPH* ciprofloxacin, *CHL* chloramphenicol, *KN* kanamycin, *PN* penicillin, *ER* erythromycin

### Enzymatic Profile

*L. plantarum* strains isolated from Oscypek and Korycinski cheeses showed weak lipolytic and proteolytic activity (excluding *L. plantarum* strains Os8, Os15, and Kor23 which showed high esterase and esterase lipase activity). Average saccharolytic activity was observed in the tested strains, especially in the case of α-galactosidase and β-galactosidase, which are technologically valuable enzymes (Table 2). High activity of β-galactosidase was observed in the case of *L. plantarum* strains Os6, Os8, Os11, Os14, Os15, Kor11, Kor12, Kor19, Kor22, and Kor23.

**Table 2:** Enzymatic profiles of the *Lactobacillus* strains

		Enzyme																			
Origin	Strain symbol	Control	Alkaline phosphatase	Esterase	Esterasel ipase	Lipase	Leucine arylamidase	Valine arylamidase	Cystine arylamidase	Trypsin	α-Chymotrypsin	Acid phosphatase	Naphthol-AS-BI-phosphohydrolase	α-Galactosidase	β-Galactosidase	β-Glucuronidase	α-Glucosidase	β-Glucosidase	N-acetyl-β-glucosaminidase	α-Mannosidase	α-Fucosidase
Oscypek	Os1	0	0	0	0	0	0	0	0	0	0	0	0	3	2	0	0	4	0	0	5
	Os2	0	1	0	0	0	5	0	0	0	0	5	5	2	2	0	0	4	0	0	1
	Os3	0	1	0	0	0	0	0	0	0	0	0	0	0	2	0	0	0	0	0	5
	Os4	0	0	0	0	0	0	0	0	0	0	0	0	2	1	0	0	0	0	0	0
	Os5	0	0	0	1	0	0	0	0	0	0	0	0	2	0	0	0	0	0	0	0
	Os6	0	0	0	1	0	0	0	0	0	0	4	0	5	5	0	0	0	0	5	0
	Os7	0	0	0	0	0	0	0	0	0	0	1	0	2	3	0	0	0	0	2	3
	Os8	0	5	5	5	0	0	0	0	0	2	4	0	5	5	0	4	5	0	5	5
	Os9	0	1	0	0	0	0	0	0	0	0	4	5	2	0	0	0	5	0	0	0
	Os10	0	0	0	0	0	0	0	0	0	1	4	0	5	0	0	5	0	0	0	0
	Os11	0	0	0	0	0	0	0	0	0	0	4	0	5	5	0	5	4	0	0	0
	Os12	0	0	0	0	0	0	0	0	0	0	0	0	1	0	0	0	1	0	0	2
	Os13	0	0	0	0	0	0	0	0	0	0	0	0	2	3	0	0	0	0	0	2
	Os14	0	5	0	0	0	0	0	0	0	0	4	3	5	5	0	5	5	0	5	0
	Os15	0	5	4	4	0	0	0	0	0	0	4	0	5	5	0	5	5	0	5	4
	Os16	0	0	5	0	0	0	0	0	0	0	0	0	0	2	0	5	5	0	5	0
	Os17	0	0	0	0	0	0	0	0	0	5	0	0	5	2	0	5	2	0	0	0
Korycinski cheese	Kor1	0	5	0	5	0	5	0	0	0	0	4	5	0	1	0	2	0	0	0	0
	Kor2	0	0	0	0	0	0	0	0	0	0	4	5	0	1	0	0	0	0	0	0
	Kor8	0	5	0	0	0	5	0	0	0	0	4	5	0	0	0	5	0	5	0	0
	Kor11	0	0	0	0	4	0	0	0	0	0	0	0	5	5	0	1	0	0	0	0
	Kor12	0	5	0	0	4	0	0	0	0	0	0	0	3	5	5	0	0	0	0	0
	Kor13	0	2	0	0	0	1	0	0	0	0	0	0	0	0	0	0	0	2	0	0
	Kor14	0	1	0	0	0	1	0	2	0	0	0	5	5	0	0	0	0	3	0	0
	Kor15	0	5	0	1	0	5	0	0	0	0	5	5	0	0	0	0	5	0	0	0
	Kor18	0	0	3	0	0	0	0	0	0	0	0	0	0	0	0	0	0	1	0	0
	Kor19	0	0	2	4	0	0	0	0	0	0	4	0	5	5	0	0	4	1	0	0
	Kor22	0	0	1	0	0	0	0	0	0	0	4	0	5	5	0	5	3	1	5	5
	Kor23	0	5	5	5	0	0	0	0	0	2	1	0	4	5	0	0	3	3	5	5

Explanatory: 0–3 negative reaction; 4–5 positive reaction

Among the tested strains isolated from both sources, the average activity of following enzymes was found: alkaline phosphatase, naphthyl-AS-BI phospholipidase, and acid phosphatase (*L. plantarum* strains Os8, Os9, Os14, Os15, Kor1, Kor2, Kor8, and Kor15). Only two of all strains produce harmful enzymes: Os17 showed the ability of α-chymotrypsin production and Kor12, which produced β-glucuronidase.

### Screening for Anti-Staphylococcal Activity

**Fig. 1:**
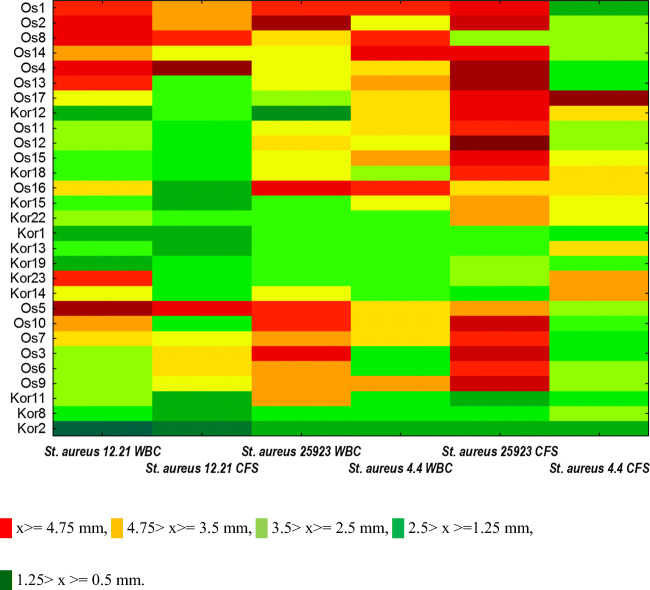
Inhibition zones map including all tested *L. plantarum* whole bacterial cultures (WBC) and cell-free supernatant (CFS) in combination with three *S. aureus* indicators strains. *x* antimicrobial activity, inhibition zone (mm).*x* ≥ 4.75 mm,  4.75 > *x* ≥ 3.5 mm,  3.5 > *x* ≥ 2.5 mm,  2.5 > *x* ≥ 1.25 mm 1.25 > x > = 0.5 mm.

All of the tested, *L. plantarum* strains exhibited the antagonistic activity against *S. aureus* strains in the experiment (Fig. 1). The anti-staphylococcal WBC activity was assessed as slight with the average diameter of inhibition growth zone of 2.82 mm ± 1.23 (Supplementary material, Figs. S1–S3). The differences (*P* < 0.05) between the WBC (average) antagonism of examined *L. plantarum* strains were found, although the influence on three *S. aureus* strains was similar (*P* > 0.05). The greatest zones of inhibition were observed for *L. plantarum* Os5 and Os2 against *S. aureus* 12.21 (4.8 mm ± 1.2; 4.2 mm ± 0.2 respectively), for *L. plantarum* Os2 and Os4 against *S. aureus* ATCC 25923 (4.3 mm ± 1.5; 4.2 mm ± 0.7 respectively) and for *L. plantarum* Os14 against *S. aureus* 4.4 (4.2 mm ± 1.2). The lowest antagonistic activity was observed for *L. plantarum* Kor2 and Kor12 against all *S. aureus* strains*.*

Differences between anti-staphylococcal effectiveness of CFS obtained from *L. plantarum* cultures against *S. aureus* strains were noted. *S. aureus* ATCC 25923 was more sensitive than *S. aureus* 12.21 and 4.4 and the observed average diameters of inhibition growth zones were 3.5 mm ± 1.3, 2.4 mm ± 1.3, and 2.6 mm ± 0.9 respectively. Significant differences (*P* < 0.05) between antagonistic activities of each *L. plantarum* strains were also observed (Fig. 1, Supplementary material, Figs. S4–S6). Low pH 4.27 ± 0.35 of tested CFSs was found.

Although, in the present study, the measured growth inhibition zones of *S. aureus* caused by WBC and CFS seem to be quite low, most of tested *L. plantarum* strains also exhibited anti-staphylococcal activities in neutralized and catalase-treated supernatant (CFN) (Supplementary material, Table S1). It was found that 11 out of 17 strains isolated from Oscypek cheese and 11 out of 12 strains isolated from Korycinski cheese slightly inhibited the growth of at least one *S. aureus* strain. The pH value of CFNs was approx. 6.5.

### Survival Ability in the Gastro-Intestinal Tract

**Fig. 2:**
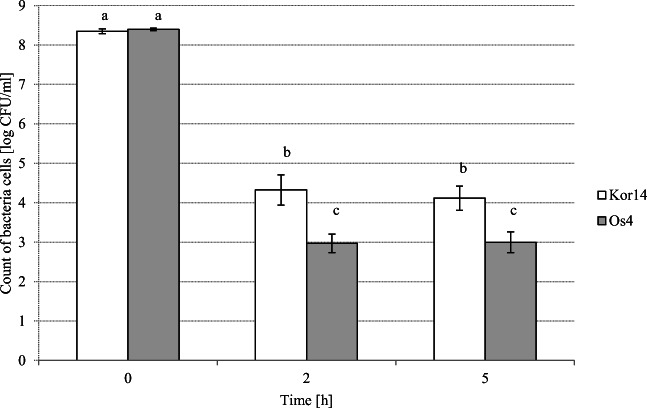
Survival of the *L. plantarum* Kor14 and Os4 strains under simulated gastro-intestinal conditions. *a*, *b*, and *c* The same letters indicate no statistically significant differences (*P* < 0.05)

The survival of two selected *L. plantarum* strains, Os4 and Kor14, during 5 h of gastro-intestinal passage are presented on Fig. 2. It was found that after 2-h incubation at gastric conditions, the number of live bacterial cells was significantly decreased (*P* < 0.05) in both samples (4–5 log units), whereas after the next 3 h of intestinal passage, the count of bacteria were stable, and at the end of passage number of *L. plantarum*, Kor14 was 4.12 ± 0.25 log CFU/ml, and it was significantly higher (*P* < 0.05) in comparison with *L. plantarum* Os4 (3.0 ± 0.23 log CFU/ml).

### Competition for Adhesion to Caco-2 Cells

**Fig. 3:**
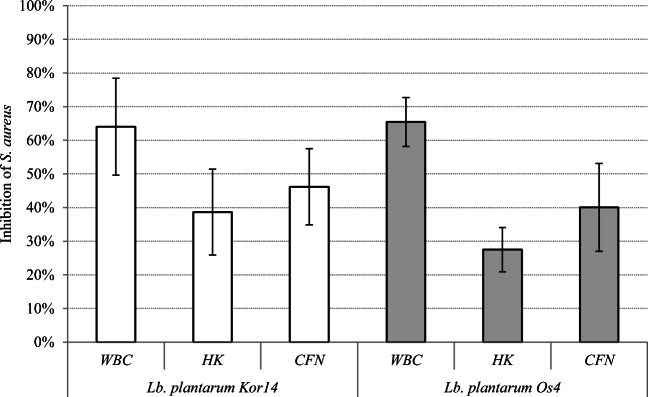
In vitro adhesion inhibition of *S. aureus* cells onto the enterocytes Caco-2 surface in tested combinations with *L. plantarum* whole bacterial cultures (WBC), *L. plantarum* heat-killed (HK) cells, and neutralized, catalase-treated cell-free supernatants (CFN) from *L. plantarum* cultures

Presence of the live *L. plantarum* Os4 and Kor14 strains (WBC) caused the strongest inhibition of *S. aureus* adherence to Caco-2 cells (65.43 ± 7.3% and 64.03 ± 14.4% respectively). The results obtained in the experiment are shown on Fig. 3. Adhesion values of *L. plantarum* in co-culture with *S. aureus* cells were 99.78 ± 6.87% for Os4 and 91.76 ± 16.75% for Kor14, which confirms the high affinity of live lactic acid bacterial cells to human enterocytes. Our results show that the adherence inhibition caused by inactivated cells (HK) occurs, but the level of the phenomenon is lower (*P* < 0.05) than the inhibition caused by live cells (WBC) of *Lactobacillus*. Presence of heat-killed *L. plantarum* Os4 and Kor14 resulted in *S. aureus* adherence inhibition to Caco-2 layer (27.51 ± 6.5% and 38.66 ± 12.8% respectively). It was observed that the inhibition of *S. aureus* adhesion in the presence of *L. plantarum* CFN was stronger comparing with HK (*P* < 0.05) but weaker comparing with WBC (*P* < 0.05). The noted inhibition of 40.06 ± 13.1% and 46.19 ± 11.3% in the presence of CFNs of *L. plantarum* Os4 and Kor14 respectively was lower (*P* < 0.05) comparing with the control. Moreover, no adherence inhibition (*P* < 0.05) caused by sterile MRS broth was observed.

### Co-Cultures in Skim Milk

**Fig. 4:**
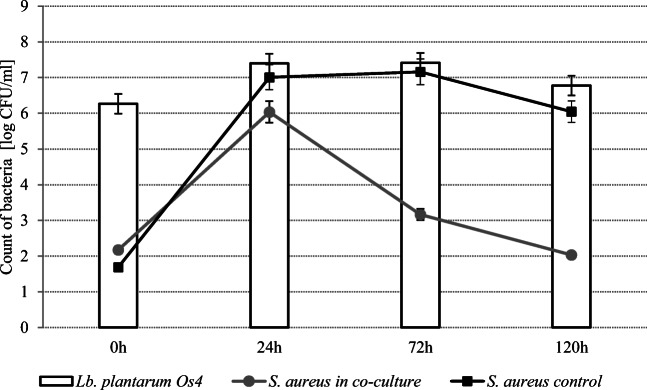
Growth of *L. plantarum* Os4 and *S. aureus* ATCC 25923 at 37 in co-culture in skim milk

**Fig. 5:**
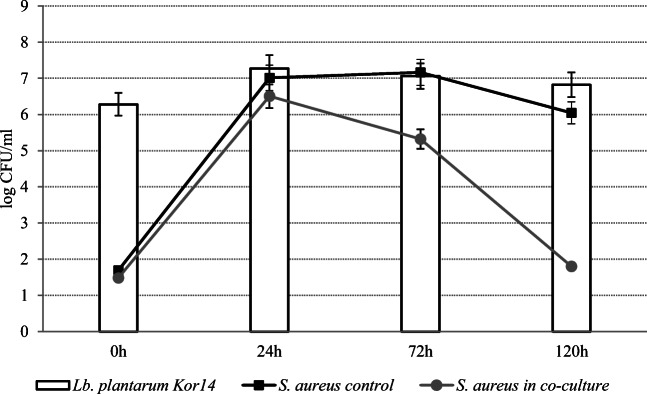
Growth of *L. plantarum* Kor14 and *S. aureus* ATCC 25923 at 37 in co-culture in skim milk

Figures 4 and 5 show the survival curves of *L. plantarum* strains in co-cultures with *S. aureus* ATCC 25923 at 37 °C. The reduction of *S. aureus* count in the samples inoculated with Os14 was greater after 72 h (*P* < 0.05) than in the case of *L. plantarum* Kor14. Generally, the number of *S. aureus* cells in skim milk samples decreased by more than 4 log CFU/ml after 120 h of incubation with *L. plantarum* Os4 and Kor14 addition. It was found that the number of *L. plantarum* Os4 was higher (*P* < 0.05) than *L. plantarum* Kor14 after 72 h of incubation.

**Fig. 6:**
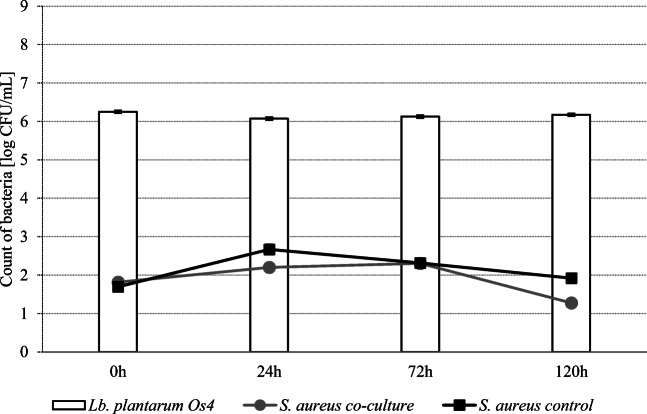
Growth of *L. plantarum* Os4 and *S. aureus* ATCC 25923 at 8 in co-culture in skim milk

**Fig. 7:**
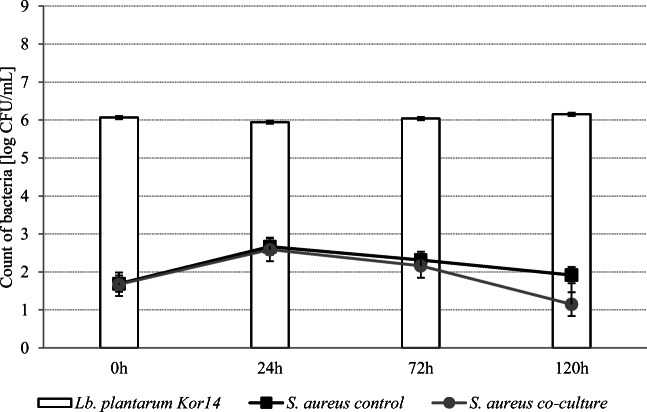
Growth of *L. plantarum* Kor14 and *S. aureus* ATCC 25923 at 8°C in co-culture in skim milk

Figures 6 and 7 show the survival curves of *L. plantarum* strains in co-culture with *S. aureus* ATCC 25923 at 8 °C. A slight increase in *S. aureus* count was observed after 24 h of incubation (0.97 log CFU/ml for the control, 0.38 log CFU/ml in the presence of *L. plantarum* Os4, and 0.91 log CFU/ml in the presence of *L. plantarum* Kor14). There were no differences between the samples (*P* < 0.05) in *S. aureus* count after 72 h of incubation, but after 120 h of incubation at 8 °C was significant (*P* < 0.05), decrease of *S. aureus* count for both the control sample and the one inoculated with *L. plantarum* strains was observed. In samples inoculated with Os4 and Kor14, *S. aureus* count decreased by more than 1 log CFU/ml comparing with the control sample at 72-h incubation time. During the whole experiment, there were no differences (*P* < 0.05) between *Lactobacillus* counts, which suggest that the cells could multiply.

## Discussion

One of the main concerns regarding technological, as well as probiotic usage of lactic acid bacteria and its safety, is resistance to antibiotics because of the possible transfer of antibiotic resistance to pathogenic bacteria. It was found that tested strains isolated from cheeses showed resistance or susceptibility to common antibiotics in regard to source of isolation. However, in some cases, the antibiotic resistance was similar. For example, resistance to vancomycin for the *L. plantarum* species is generally considered to be an intrinsic resistance that is associated with the construction of the bacterial cell wall. Resistance to streptomycin could be the reason of chromosomal mutations and therefore does not represent a potential risk of transferability [18]. According to EFSA Scientific Opinion (The FEEDAP Panel) [14] where all strains within a given taxonomic group show phenotypic resistance to an antimicrobial, such resistance can be considered intrinsic to the taxonomic group. Strains carrying intrinsic resistance present a minimal risk for horizontal spread and can be used as a feed additive [14]. On the other hand, susceptibility to gentamicin for *L. plantarum* strains has been observed previously in the study of Guo et al. [19]. Various studies have shown that the levels of susceptibility of *Lactobacillus* spp. to antibiotics were species-dependent or even strain-dependent. For example, in the Plessas et al. [20] study, strains isolated from Feta-type cheese have been demonstrated to be resistant to vancomycin, tetracycline, and erythromycin. Also, other authors confirmed *Lactobacillus* genus resistance to vancomycin and tetracycline [21]. The widespread incidence of antibiotic resistance in dairy food isolates is caused by the extensive use of antibiotics over the past few years in both animals and humans feeding [22].

The results obtained in the present study confirm the safety of tested *L. plantarum* strains in regard to antibiotic resistance; however, future study should be focused on identification and localization of antibiotic resistance genes. In the absence of information on the genetic nature of a demonstrated resistance, the strain should not be used as a feed additive [14].

The enzymatic profile of LAB used in cheese production is one of the factors which affect cheese flavor the most [21]. Lipolytic and proteolytic activity of *Lactobacillus* is species related, which was previously reported [23] and was observed in our study. Production of β-galactosidase is important for the production of dairy products and for consumers of dairy products who have lactose intolerance [24]. High β-galactosidase activity was observed in several of our strains (Os6, Os8, Os11, Os14, Os15, Kor11, Kor12, Kor19, Kor22, and Kor23). Among the *L. plantarum* strains, the average activities of phosphatase enzymes were found, which are the essential enzymes for the hydrolysis of phosphopeptides prevalent in cheese ripening [21]. From the safety point of view, it is important that bacterial strains do not produce harmful enzymes, i.e., α-chymotrypsin, β-glucuronidase, and *N*-acetyl-β-glucosaminidase, as they are known to be potential mediators of colon carcinogenesis [25]. Our results indicate that the enzymatic activity of tested strains should be considered as safe, except for Os17 and Kor12, which produced harmful enzymes.

An important element in the assessment of probiotic bacteria is their antimicrobial properties. All of strains tested in this study were previously assessed for their antagonistic activity against other pathogens such as *L. monocytogenes*, *S. enteritidis*, and *E. coli* as well as two food isolates *Bacillus subtilis* and *Enterococcus faecium* strains [11].

In the present study, anti-staphylococcal effectiveness of whole bacterial culture (WBC), cell-free supernatant (CFS), and neutralized, catalase-treated cell-free supernatant (CFN) was examined. The WBC contained live *L. plantarum* cells and all potential metabolites such as organic acids, hydrogen peroxide, and peptide substances. Anti-staphylococcal activity of CFSs was investigated in order to assess the influence of antimicrobial metabolites of *L. plantarum* cells, without the influence of live cells of bacteria. CFN used in the tests did not contain live or killed *Lactobacillus* cells and also organic acids and hydrogen peroxide activity were blocked by neutralization. The fact that neutralization of the supernatant caused the disappearance of antimicrobial activity for some of the strains, and its significant reduction for others, confirms that the low pH and organic acids present in CFS and WBC are the most important antimicrobial agents. This finding is in agreement with other studies [11, 15, 26].

Well diffusion method results of the present study showed generally much lower antagonistic activity of *L. plantarum* strains against *S. aureus* than for the other previously tested pathogens. In previous studies it was found that the *L. plantarum* Os1-Os17 strains exhibited strong anti-*Listeria* properties. In turn, *L. plantarum* strains isolated from Korycinski cheeses showed stronger antagonistic activity against *E. coli* in comparison with *L. plantarum* strains isolated from Oscypek [11]. Some other researchers did not observe the anti-staphylococcal activity of *L. plantarum* [27–29]. Arena [10] also assessed the antagonistic activity of *L. plantarum* strains as rather lower against *S. aureus* than *L. monocytogenes*, *S. enteritidis*, and *E. coli*. In turn, in other study, *S. aureus* growth inhibition zones were reported similar to the ones for other pathogens such as *E. coli*, *Listeria innocua*, and *Salmonella typhimurium* [7]. Karska-Wysocki et al. [30] reported similar to our study diameters of MRSA growth inhibition zones, which were enlarged when mixtures of few LAB strain cultures were tested. In turn, huge diversity of *S. aureus* growth inhibition zones ranging from 0.5 to 10 mm when plating with different strains of *L. plantarum* was observed by [10]. This confirms strain-dependent antimicrobial activity of lactic acid bacteria, demonstrated already by many studies [10–12, 29].

The tested *L. plantarum* strains have shown broad spectrum of antimicrobial activity, which in connection with present findings can be useful in food technology. Basing on our current results, we decided to select two most promising *L. plantarum* strains and test their possibility to compete for adherence to humans cells in vitro as well as in situ anti-staphylococcal activity in food matrix. *L. plantarum* Os4 and Kor14 strains have been chosen, based on average anti-staphylococcal activity for WBC and CFS, and according to their enzymatic activity profile as well as antibiotic resistance.

Although tested *L. plantarum* strains exhibited promising anti-bacterial properties, they should be at least resistant for gastro-intestinal conditions in the case of application as probiotic cultures. Generally, *L. plantarum* is known to be resistant to mucin, low gastric pH, and bile salts; however these features are in most cases strains related [31]. Similar results have been previously reported by others [32, 33], who highlighted the significant impact of low pH on the survival of lactobacilli. On the other hand, the stabilization of viability, observed under intestinal conditions, suggests that the tested *L. plantarum* strains could reach the intestine with significant concentrations of live cells, even after exposure to harmful acid challenge.

Many studies have shown that lactic acid bacteria compete with pathogens for adhesion to enterocytes which is an important feature of probiotic strains [1, 8, 9]. In the present study, it was decided the adhesion competition to Caco-2 cells as a good model for anti-staphylococcal activity test should be assessed. Strong adhesion competition of *L. plantarum* Os4 and Kor14 strains with *S. aureus* suggest that these strains are able to adhere, at least temporary, to the intestinal tract.

Many studies have shown that *Lactobacillus* adherence to Caco-2 varies among the genus [8, 34]. Some researchers have argued that even killed LAB cells can inhibit the adherence of pathogens to intestinal surfaces [35, 36]. In our study, also moderate adhesion inhibition caused by HK cells occurred. Inhibition of pathogen adhesion to Caco-2 cells may result from the biofilm formation by heat-killed *Lactobacillus* cells and the influence of bacterial metabolites on enterocyte layer [37]. Stronger adhesion inhibition of *L. plantarum* CFN was exhibited by Os4 and Kor14, which was probably caused by antimicrobial factors presented in supernatant. In other study, neutralized supernatant from *Bifidobacterium* cultures in MRS broth inhibits the adherence of *Clostridium difficile* in a similar scenario [38]. MRS did not cause adherence inhibition, which supports the thesis that tested CFNs containing other anti-adherent substances produced by examined *L. plantarum* strains.

Taking into account that the investigated LAB strains were isolated from dairy products, the skim milk matrix was chosen for the in situ studies. In the experiment, the inoculation with the *S. aureus* in approx. 10^2^ CFU/ml and at the same time with *L. plantarum* cells (10^6^ CFU/ml) reflected the situation of cross-contamination that may happen during the production of fermented milk products. The pathogenic effects of *S. aureus* are connected with enterotoxin production in food matrix. It has been proved that 5 × 10^5^ cells of enterotoxin-producing *S. aureus* strains per ml is a threshold value resulting in the production of enterotoxin [39, 40]. Therefore, the key prevention should be to eliminate any factors and conditions that enhance the growth of the pathogen and the production of enterotoxin [41].

It has been found that *L. plantarum* strains Os4 and Kor14 successfully inhibited the growth of *S. aureus* at 37 °C in milk, but the dynamic of action was strain-dependent. Different growth kinetics of *L. plantarum* Os4 strain may be the reason for faster *S. aureus* count reduction in co-cultures in comparison with Kor14. In another study, *S. aureus* count decreased below the detection limit after 72 h of incubation when milk was inoculated with 3 log CFU/ml of both *L. plantarum* and *S. aureus* [42]. It was also shown that presence of *S. aureus* in co-culture with some *L. plantarum* strains slows down the pH lowering in the matrix, which could be the reason of overdue the effect of exhibited anti-staphylococcal efficiency [40].

The influence of the environmental conditions for the antimicrobial effectiveness of tested *L. plantarum* strains was also examined by the experiment conducted in a lower temperature. Slight inhibition of *S. aureus* growth was observed after 120 h of incubation. The temperature of 8°C was chosen to simulate the refrigeration conditions. Although there are many studies showing that the *L. plantarum* growth is inhibited in temperatures below 15 °C, the minimum temperature for *S. aureus* growth is 7 °C [43]. For example, the lag phase of *Lactobacillus rhamnosus* GG growth in 8 °C was long and lasted for 17.99 h. Moreover, this phenomenon results in a very slow lowering of the pH of the environment [44]. Many studies also show that in lower temperatures (e.g., 10–15 °C), synthesis of bacteriocins and other antimicrobial compounds with maximum yield was observed [45–47]. Lowering of *S. aureus* count in samples inoculated with *L. plantarum* Os4 and Kor14 strains can therefore be associated with other factors than organic acids, like bacteriocins. Moreover, used in this study, a high ratio of *L. plantarum* to *S. aureus* may facilitate *Lactobacillus* cells to the competition for nutrients in tested biological niche.

In conclusion, *L. plantarum* strains isolated from regional cheeses should generally be considered as safe; however, their antibiotic resistance was found to be source of isolation related. Most of *L. plantarum* strains isolated from the Oscypek were resistant to ciprofloxacin, whereas strains isolated from the Korycinski cheese were resistant to penicillin and erythromycin. According to the enzymatic profile study, the following strains should be excluded from further studies: Os17, which showed the production of α-chymotrypsin and Kor12, which produced β-glucuronidase.

All tested *L. plantarum* strains exhibited anti-staphylococcal activity, and almost all CFN of tested strains exhibit slight anti-staphylococcal activity. Chosen *L. plantarum* Os4 and Kor14 strains were able to survive in simulated human gastro-intestinal tract. *S. aureus* adhesion to Caco-2 cells was inhibited in the presence of *L. plantarum* live bacterial cells, as well as and dead bacterial cells and CFN. Therefore, tested *L. plantarum* strains of food origin could successfully compete with this pathogen in human gut. Moreover, the decrease in *S. aureus* counts in the milk matrix resulting from the presence of *L. plantarum* Os4 and Kor14 both at 37 and 8 °C confirms that these strains or their metabolites could be potentially used in the food industry as protective cultures to extend the shelf life of foodstuffs, in particularly fermented milk products.

Basing on our study, we recommend two strains of *L. plantarum* Os4 and Kor14 for use in food technology. Further studies are needed to evaluate the *L. plantarum* bacteriocin production and also to develop an efficient method for *L. plantarum* Os4 and Kor14 application as probiotic and protective cultures.

## Electronic Supplementary Material

ESM 1 (DOCX 354 kb)
