# Supplementary material for: Lactobacillus plantarum Strains Isolated from Polish Regional Cheeses Exhibit Anti-Staphylococcal Activity and Selected Probiotic Properties
Source: Probiotics Antimicrob Proteins. 2019 Aug 28;12(3):1025–38. doi: 10.1007/s12602-019-09587-w (PMC7456411; doi:10.1007/s12602-019-09587-w)
Supplement: Supplementary file 1 — (DOCX 354 kb) [file 12602_2019_9587_MOESM1_ESM.docx]

**Supplementary material**


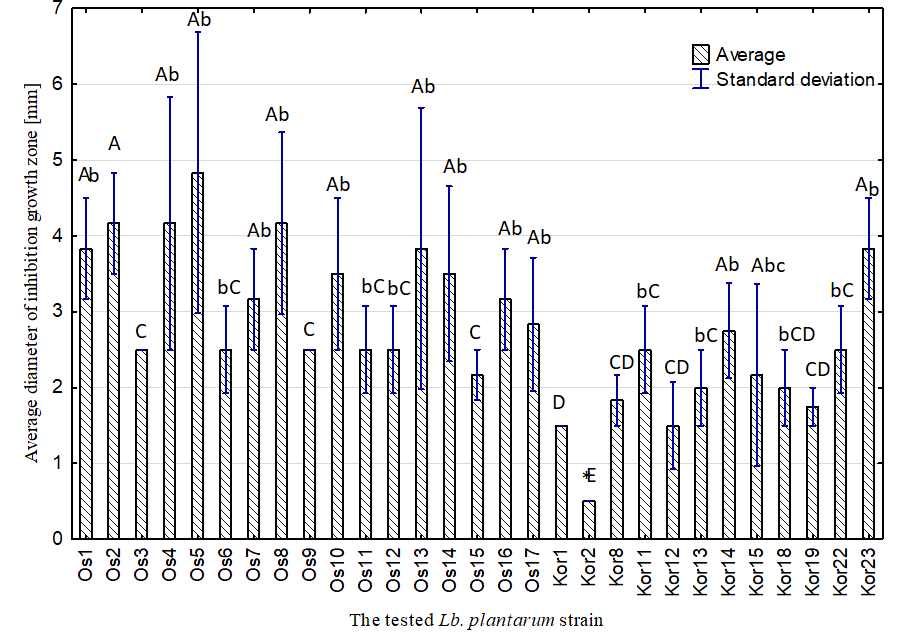


Fig. S1. Antagonistic activity of tested WBC *Lb. plantarum* strains against *S. aureus* 12.21. Different letters indicate different groups (P<0,05) by ANOVA.


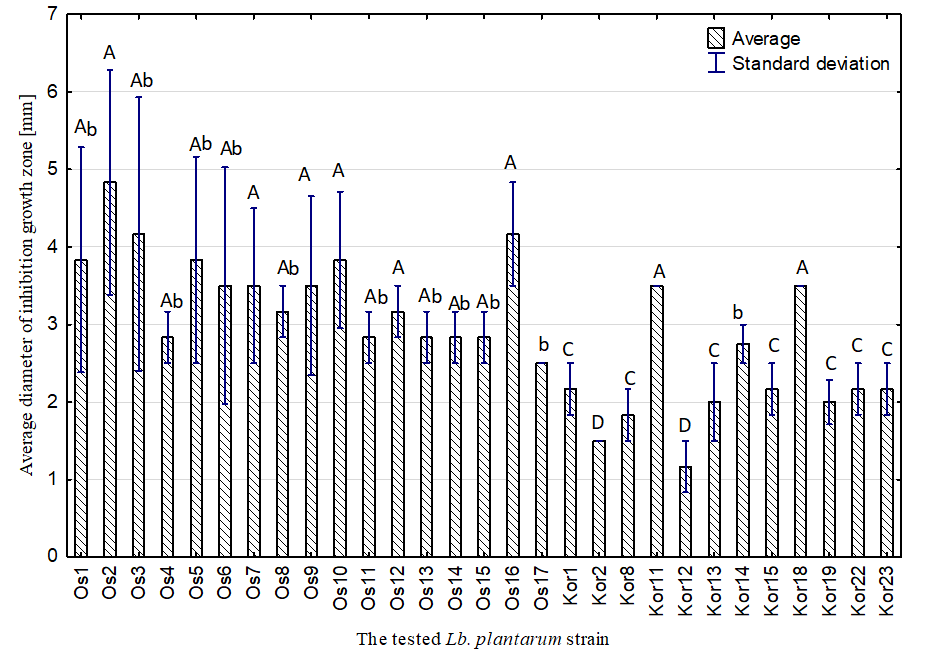


Fig. S2. Antagonistic activity of tested WBC *Lb. plantarum* strains against *S. aureus* ATCC 25923. Different letters indicate different groups (P<0,05) by ANOVA.


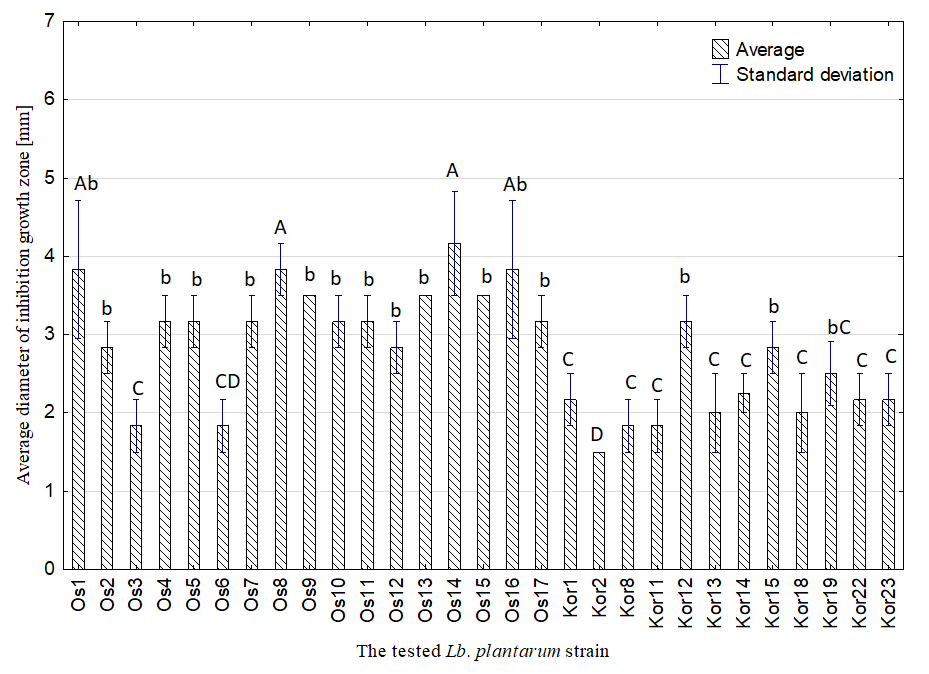


Fig. S3. Antagonistic activity of tested WBC *Lb. plantarum* strains against *S. aureus* 4.4. Different letters indicate different groups (P<0,05) by ANOVA.


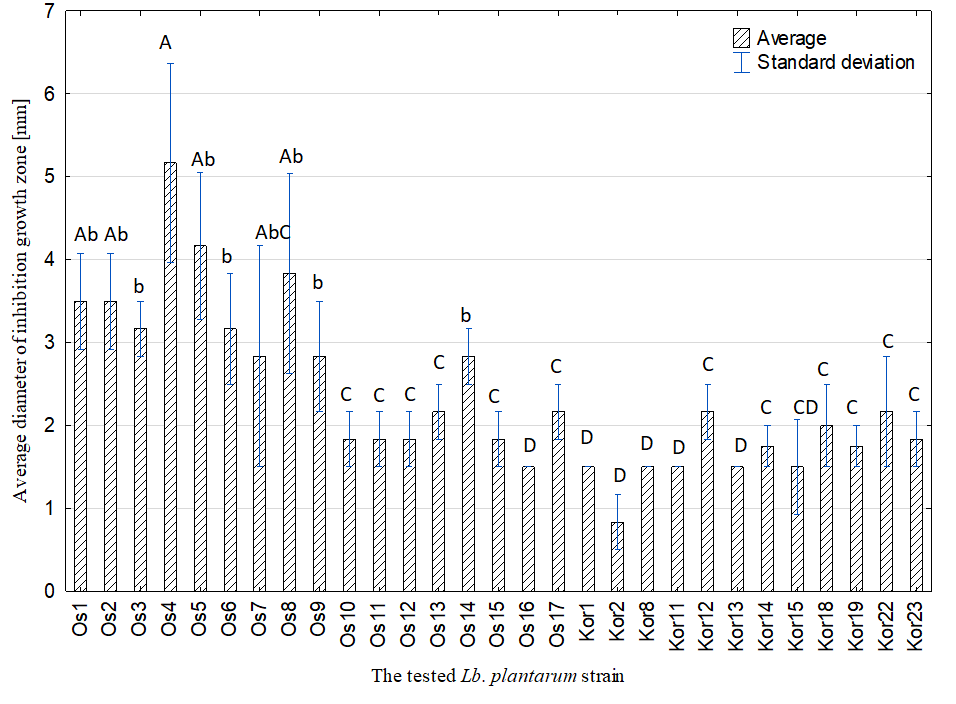


Fig. S4. Antagonistic activity of tested CFS *Lb. plantarum* strains against *S. aureus* 12.21. Different letters indicate different groups (P<0,05) by ANOVA.


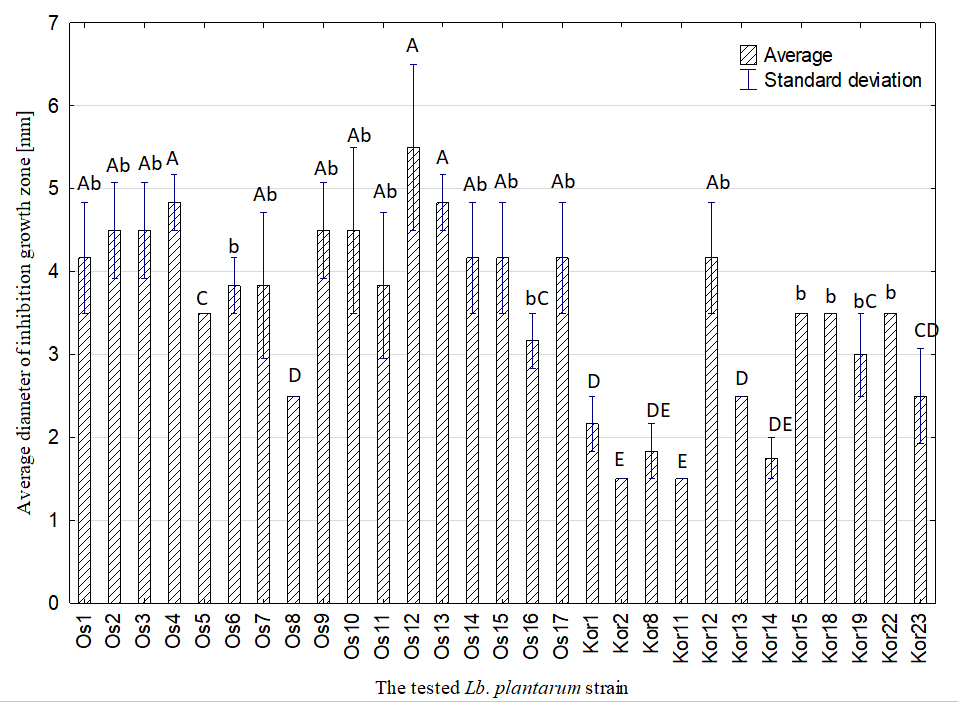


Fig. S5. Antagonistic activity of tested CFS *Lb. plantarum* strains against *S. aureus* ATCC 25923. Different letters indicate different groups (P<0,05) by ANOVA.


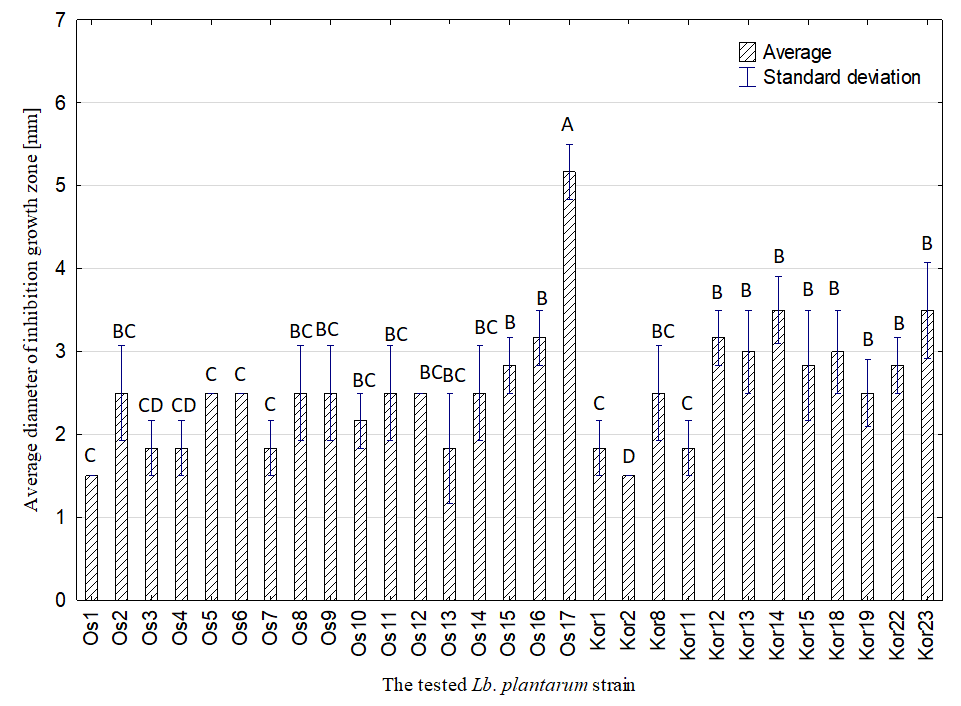


Fig. S6. Antagonistic activity of tested CFS *Lb. plantarum* strains against *S. aureus* 4.4. Different letters indicate different groups (P<0,05) by ANOVA.

**Supplementary material**

Table S1. Anti-staphylococcal activity of tested CFNs *Lb. plantarum* cultures against *S. aureus*

| *Lb. plantarum* strain | S. aureus  12.21 | S. aureus ATCC 25923 | S. aureus  4.4 |
| --- | --- | --- | --- |
| Os1 | - | + | + |
| Os2 | + | + | + |
| Os3 | - | - | - |
| Os4 | + | + | + |
| Os5 | + | + | + |
| Os6 | - | - | - |
| Os7 | - | - | - |
| Os8 | + | + | + |
| Os9 | + | + | + |
| Os10 | - | + | + |
| Os11 | - | + | - |
| Os12 | - | - | - |
| Os13 | + | - | + |
| Os14 | + | + | - |
| Os15 | + | + | - |
| Os16 | - | - | - |
| Kor1 | - | - | - |
| Kor2 | + | + | + |
| Kor8 | + | + | + |
| Kor11 | + | - | - |
| Kor13 | + | + | + |
| Kor14 | + | + | + |
| Kor15 | + | - | + |
| Kor18 | + | + | - |
| Kor19 | - | + | + |
| Kor22 | - | + | + |
| Kor23 | + | + | - |

Explanatory: ‘+’ – measured diameter of inhibition growth zone < 1mm (n=3); ‘-‘ – no observed *S. aureus* inhibition growth zone.
